# Enhancing Writing Self-Regulation and Metacognitive Awareness Through Structured Free Writing: A Mixed-Methods Study

**DOI:** 10.3390/jintelligence14070135

**Published:** 2026-07-02

**Authors:** Nurullah Aykaç

**Affiliations:** Education Faculty, Dicle University, Diyarbakir 21280, Turkey; nurullah.aykac@dicle.edu.tr

**Keywords:** self-regulated learning, metacognition, writing self-regulation, individual differences, cognitive processes, writing performance, higher education

## Abstract

Drawing on research on self-regulated learning, metacognition, and cognitive aspects of writing, this study examines whether structured free writing can function as a scaffold for developing writing self-regulation, affective engagement, and genre-based performance. While free writing has traditionally been conceptualized as a creativity-enhancing or pre-writing activity, its role in fostering self-regulatory and metacognitive processes associated with intelligent performance remains underexplored. A convergent parallel mixed-methods design was employed with preservice teachers enrolled in a seven-week writing workshop in higher education. Quantitative data included validated measures of writing self-regulatory efficacy and attitudes toward free writing, along with analytic assessments of informative and narrative writing performance. Qualitative data were obtained through semi-structured interviews examining participants’ regulation of their writing processes. Findings indicate significant improvements in self-regulatory efficacy, more adaptive attitudes toward writing, and gains in genre-specific performance. Qualitative results further suggest that structured free writing supports metacognitive awareness, reduces writing-related anxiety, and enhances sustained cognitive engagement. By conceptualizing free writing as a mechanism for developing self-regulatory capacities underlying intelligent behavior, this study contributes to research on individual differences in learning and highlights the integration of cognitive and affective processes in academic performance.

## 1. Introduction

Language serves as a fundamental medium through which individuals construct, express, and negotiate meaning, enabling the communication of knowledge, emotions, and ideas. Effective communication depends on the coordinated development of receptive and productive language skills—listening, speaking, reading, and writing—which evolve in an interconnected and recursive manner. While listening and reading provide linguistic input and conceptual resources, speaking and writing function as productive modes through which individuals transform internalized knowledge into meaningful expression. Among these skills, writing occupies a distinctive position, as it requires not only linguistic competence but also higher-order cognitive processes such as planning, organizing, revising, and evaluating ideas (Flower & Hayes, 1981; Graham et al., 2012).

Writing is therefore widely recognized as a complex, multidimensional, and production-oriented skill, integrating cognitive, metacognitive, and affective components. Unlike other language skills, writing demands sustained attention, conscious regulation, and deliberate decision-making throughout the composing process. For this reason, it is often considered one of the most challenging skills to acquire and teach effectively (Atasoy, 2021; Türkben, 2021). At the same time, writing functions both as a means of learning and as a learning outcome, positioning it as a central component of formal education across disciplines. Beyond academic contexts, writing plays a crucial role in everyday life, professional communication, and civic participation. Competence in writing is increasingly viewed as essential for employability, lifelong learning, and social engagement (Festas et al., 2015; Yu & Lee, 2024; Mitchell et al., 2025). However, research consistently shows that many learners experience difficulties in expressing their thoughts in writing, often accompanied by negative attitudes, low motivation, and writing anxiety. When students perceive writing as demanding, restrictive, or evaluative, they are less likely to engage actively in the writing process, which in turn hinders skill development (Aktaş & Akyol, 2020; Daly & Miller, 1975).

One factor closely associated with successful writing development is self-regulation. Self-regulated writers are able to set goals, plan their texts, monitor their progress, revise strategically, and sustain motivation despite challenges (Zimmerman, 2000; Schunk & Zimmerman, 2007). Empirical studies indicate that students who demonstrate stronger self-regulatory efficacy tend to produce higher-quality texts and show greater persistence in writing tasks (Graham et al., 2012; Zhang & Zhang, 2022). Nevertheless, self-regulation does not develop automatically; it requires instructional environments that encourage autonomy, reflection, feedback, and iterative practice.

In this regard, writing workshops have emerged as pedagogical spaces that support writing development through student-centered, process-oriented, and collaborative practices. Writing workshops emphasize experimentation, peer interaction, feedback, and revision, positioning learners as active participants rather than passive recipients of instruction (Berne, 2009; Kohlloffel, 2025). Within such environments, learners are encouraged to explore their voices, reflect on their writing processes, and engage in meaningful dialogue with peers and instructors.

A key practice frequently incorporated into writing workshops is free writing, which allows learners to write without strict constraints related to topic, genre, or evaluation. Free writing has been shown to reduce anxiety, enhance creativity, and foster more positive attitudes toward writing by shifting the focus from correctness to expression (Elbow, 1973; Alharthi, 2021). When learners are given control over content and expression, they are more likely to engage authentically with writing tasks, which can serve as a foundation for developing self-regulatory strategies. Despite the documented benefits of writing workshops, free writing, and self-regulated learning, research examining their combined influence remains limited, particularly in the context of teacher education. Preservice teachers represent a critical population, as their beliefs, attitudes, and competencies related to writing are likely to shape their future instructional practices. Understanding how writing workshops influence preservice teachers’ writing attitudes and self-regulatory efficacy is therefore essential for designing effective writing pedagogy in teacher preparation programs.

Responding to this need, the present study investigates the effects of a seven-week writing workshop implemented at a public university in Türkiye on undergraduate preservice teachers’ free writing attitudes, self-regulatory efficacy for writing, and writing performance across informative and narrative genres. Focusing on the Turkish higher education context is particularly relevant because writing workshops remain relatively underexplored within teacher education programs in the region, despite increasing recognition of the importance of self-regulated writing for future teachers. Employing a convergent parallel mixed-methods design, the study integrates quantitative measures with qualitative insights to provide a comprehensive account of how writing workshops influence both measurable outcomes and participants’ experiences as developing writers and future teachers of writing.

## 2. Literature Review

### 2.1. Writing as a Cognitive, Affective, and Self-Regulated Process

Writing is widely conceptualized as a complex, recursive process that integrates cognitive, metacognitive, and affective dimensions. Early cognitive models of writing, most notably the framework proposed by Flower and Hayes (1981), emphasized writing as a problem-solving activity involving planning, translating, and reviewing. Subsequent models expanded this view by highlighting the dynamic interplay between writers’ goals, prior knowledge, motivation, and the social context in which writing occurs (Hayes, 2012; Graham et al., 2012).

From an educational perspective, writing is not merely a linguistic skill but a learning tool through which individuals construct knowledge, reflect on experience, and engage in disciplinary thinking. However, research consistently indicates that learners often experience writing as cognitively demanding and emotionally taxing, particularly when instructional practices emphasize correctness and product over process (Daly & Miller, 1975; Bruning & Horn, 2000). Negative writing experiences can lead to avoidance behaviors, low self-efficacy, and resistance to writing tasks, ultimately constraining learners’ development. These challenges emphasize the importance of instructional approaches that address not only the cognitive demands of writing but also learners’ attitudes, beliefs, and self-regulatory capacities. In this regard, self-regulated learning theory offers a robust framework for understanding how learners manage the complexities of writing.

The literature reviewed in this study is organized around four interrelated themes that collectively provide the theoretical and empirical foundation for the research. These themes were selected deductively based on the study’s conceptual framework and research objectives rather than through a formal thematic analysis of the literature. Specifically, the review focuses on (a) self-regulated learning and writing, which provides the primary theoretical lens for understanding writing development; (b) writing workshops as instructional environments, which constitute the pedagogical intervention examined in the study; (c) free writing and writing attitudes, which relate to the affective dimension of writing development; and (d) writing instruction in preservice language teacher education, which defines the educational context of the research. The final subsection synthesizes these strands of literature to identify the research gap and establish the rationale for the present study.

### 2.2. Self-Regulated Learning and Writing

Self-regulated learning (SRL) refers to learners’ active engagement in setting goals, selecting strategies, monitoring progress, and regulating motivation and behavior during learning tasks (Zimmerman, 2000; Schunk & Zimmerman, 2007). Within writing research, self-regulation has been associated with effective planning, strategic revision, persistence, and higher-quality text production (Graham et al., 2012; Festas et al., 2015).

Self-regulated writing involves three interrelated phases: forethought (goal setting and planning), performance (strategy use and self-monitoring), and self-reflection (evaluation and adaptation). Writers who demonstrate strong self-regulatory efficacy are more likely to approach writing tasks strategically and to view challenges as manageable rather than threatening (Zimmerman & Risemberg, 1997; Fernandez & Guilbert, 2024). Conversely, writers with low self-regulatory beliefs often struggle to sustain effort, particularly when faced with complex or open-ended writing tasks.

Empirical studies have shown that self-regulation can be enhanced through instructional interventions that promote autonomy, reflection, and feedback (Chen et al., 2025; Graham & Harris, 2018; M. F. Teng et al., 2022; Fan & Wang, 2024; Hwang, 2025). However, much of this research has focused on explicit strategy instruction or isolated classroom activities. Less attention has been paid to instructional environments, such as writing workshops, which naturally embed opportunities for self-regulation through iterative practice, peer interaction, and reflective dialogue.

### 2.3. Writing Workshops as Instructional Environments

Writing workshops are grounded in constructivist and socio-cognitive theories of learning, emphasizing learner agency, process-oriented instruction, and social interaction. Typically, writing workshops involve cycles of planning, drafting, peer feedback, revision, and reflection, allowing learners to engage with writing as an evolving process rather than a static product (Berne, 2009; Graham & Perin, 2007).

Research suggests that writing workshops contribute positively to learners’ writing quality, engagement, and motivation by fostering a supportive and collaborative learning climate (Jasmine & Weiner, 2007; Kohlloffel, 2025). Within such environments, learners are encouraged to experiment with ideas, receive formative feedback, and develop a sense of ownership over their writing. These features align closely with the principles of self-regulated learning, as learners are required to make strategic decisions, monitor progress, and revise based on feedback.

Despite these benefits, studies examining writing workshops have often prioritized performance outcomes, such as text quality or genre mastery, while paying comparatively less attention to learners’ affective experiences and self-regulatory development. This gap is particularly evident in higher education settings, especially within preservice teacher education programs, where writing workshops have been studied less systematically than in primary and secondary education contexts. Although writing competence and self-regulation are essential professional skills for future teachers, relatively little research has explored how writing workshop–based interventions influence preservice teachers’ attitudes toward writing, self-regulatory efficacy, and writing development during their undergraduate teacher preparation.

### 2.4. Free Writing and Writing Attitudes

Free writing, popularized by Elbow (1973), is a pedagogical practice that encourages learners to write continuously without concern for correctness, structure, or evaluation. By removing external constraints, free writing aims to reduce writing anxiety, stimulate idea generation, and promote fluency. Empirical research indicates that free writing can help learners develop more positive attitudes toward writing by reframing it as a means of expression rather than assessment (Alharthi, 2021; Atasoy, 2021).

Writing attitudes encompass learners’ beliefs, emotions, and dispositions toward writing tasks. Positive writing attitudes have been associated with greater engagement, persistence, and willingness to revise, whereas negative attitudes are linked to avoidance and reduced effort (Bruning et al., 2013; Aktaş & Akyol, 2020). Instructional practices that emphasize freedom, choice, and expression such as free writing can therefore play a critical role in shaping learners’ affective orientations toward writing.

However, while free writing has been widely discussed in practitioner-oriented and composition literature as a means of promoting fluency, creativity, and idea generation (Elbow, 1973; Alharthi, 2021), empirical studies examining its role within structured instructional models such as writing workshops remain comparatively limited, particularly in higher education settings. Moreover, the relationship between learners’ attitudes toward free writing and their self-regulatory efficacy has received limited empirical attention, despite theoretical perspectives suggesting that motivation, affective engagement, and positive writing experiences are important foundations for the development of self-regulated learning behaviours (Bruning & Horn, 2000; Zimmerman, 2000; L. S. Teng, 2022).

### 2.5. Writing Instruction in Language Teacher Education

Teacher education represents a critical context for writing research, as preservice teachers are expected to develop both personal writing competence and pedagogical knowledge related to writing instruction. Previous research has shown that preservice teachers’ attitudes toward writing, beliefs about themselves as writers, and writing identities influence both their own writing development and their future approaches to writing instruction (Hall & Grisham-Brown, 2011; Morgan, 2010). Studies further indicate that many preservice teachers enter teacher education programs with limited confidence in their writing abilities and often require sustained opportunities to engage in authentic writing experiences in order to develop stronger writer identities and greater instructional confidence (Morgan, 2010; Cremin & Oliver, 2017). Consequently, researchers have increasingly emphasized the importance of teacher education experiences that position preservice teachers as active writers who engage in reflection, collaboration, and meaningful writing practice.

Within language teacher education, writing occupies a particularly important role because future language teachers are expected not only to demonstrate proficiency in written communication but also to teach writing effectively in their future classrooms. Research in first-language (L1) teacher education suggests that preservice language teachers’ experiences as writers influence their beliefs about writing instruction, their confidence in teaching writing, and the pedagogical approaches they later adopt (Hall & Grisham-Brown, 2011; Morgan, 2010; Cremin & Oliver, 2017). Consequently, teacher education programs increasingly emphasize opportunities for authentic writing, reflection, and engagement with process-oriented writing pedagogy.

Despite this growing body of research, relatively little attention has been devoted to examining how writing workshop–based interventions incorporating structured free writing influence preservice teachers’ writing attitudes, self-regulatory efficacy, and writing performance simultaneously. Furthermore, few studies have employed mixed-methods approaches to explore both the measurable outcomes and lived experiences associated with these instructional practices. Addressing these gaps, the present study investigates the combined cognitive, affective, and performance-related outcomes of a writing workshop implemented with preservice teachers.

### 2.6. Research Gap and Contribution of the Study

Although a substantial body of research has examined writing instruction, writing attitudes, and self-regulated learning separately, empirical studies that integrate these constructs within an authentic instructional context remain limited, particularly in teacher education. Existing research on writing workshops has predominantly focused on their impact on writing performance or motivation (Berne, 2009; Graham & Perin, 2007; Jasmine & Weiner, 2007), while studies on self-regulation in writing have largely investigated strategy instruction or individual cognitive processes in controlled classroom settings (Zimmerman, 2000; Graham et al., 2012; Cheng & Zhang, 2021).

What remains underexplored is how writing workshop–based instruction shapes preservice teachers’ affective orientations toward writing (free writing attitudes) and their self-regulatory efficacy simultaneously, and how these changes are reflected in actual written products across different text genres. In particular, free writing, despite its documented benefits for reducing anxiety and fostering creativity (Elbow, 1973; Alharthi, 2021), has rarely been examined as a pedagogical bridge between writing attitude and self-regulation in structured higher education contexts.

Furthermore, while self-regulated writing has been widely recognized as a critical competence for academic success and lifelong learning (Schunk & Zimmerman, 1998; Festas et al., 2015), empirical evidence documenting how instructional environments such as writing workshops support the internalization of self-regulatory processes among future teachers remains scarce. This gap is especially salient in preservice teacher education, where future teachers are expected to develop both their own writing competence and the pedagogical knowledge necessary to support writing development in their future classrooms. Research suggests that preservice teachers’ beliefs, confidence, and experiences as writers influence their subsequent approaches to writing instruction and the opportunities they provide for student writing (Hall & Grisham-Brown, 2011; Morgan, 2010; Graham et al., 2012).

### 2.7. Research Questions

Grounded in self-regulated learning theory and process-oriented writing pedagogy, this study investigates the effects of a writing workshop incorporating free-writing activities on the writing-related outcomes of preservice Turkish language teachers. The primary aim is to examine how participation in the workshop influences writing attitudes, self-regulatory efficacy, and participants’ perceptions of their writing development. Unlike many other teacher education disciplines, language teacher education places a particular emphasis on writing because future language teachers are expected both to develop advanced writing competence and to teach writing effectively in their future classrooms. Situated within the context of first-language (L1) writing education, the study employs a convergent parallel mixed-methods design and addresses the following research questions:

RQ1. What is the effect of a writing workshop incorporating free writing activities on preservice teachers’ attitudes toward free writing?

RQ2. What is the effect of the writing workshop on preservice teachers’ self-regulatory efficacy in writing?

RQ3. To what extent does participation in the writing workshop influence preservice teachers’ writing performance across different text genres (informative and narrative)?

RQ4. How do preservice teachers describe their experiences of free writing, self-regulation, and writing development within the context of the writing workshop?

RQ5. How do the qualitative findings converge with the quantitative results in explaining the impact of the writing workshop on pre-service teachers’ writing attitudes, self-regulatory efficacy, and writing performance?

## 3. Method

### 3.1. Research Design

The present study adopted a convergent parallel mixed-methods design (Creswell & Plano Clark, 2018), integrating both quantitative and qualitative data to provide a comprehensive understanding of the effects of the writing workshop. The quantitative strand was based on a one-group pre-test–post-test design (Campbell & Stanley, 1963) for the attitudinal and self-regulation scales, which enabled the measurement of changes in preservice teachers’ free writing attitudes and self-regulatory efficacy for writing following the instructional intervention. In addition, participants’ writing performance was assessed at the end of the workshop through rubric-based evaluations of informative and narrative texts, providing a summative measure of their written expression skills. Complementing this, the qualitative strand employed semi-structured interviews, allowing for in-depth exploration of participants’ perceptions, experiences, and challenges during the workshop process. Employing both strands concurrently allowed for the triangulation of findings and the enrichment of the interpretation of results.

### 3.2. Participants and Data Collection Process

The participants of this study were 26 preservice Turkish language teachers enrolled in the Faculty of Education at Dicle University during the 2024–2025 academic year. All participants voluntarily attended the seven-week writing workshop and agreed to participate in the study. As future teachers of the Turkish language, they represented an appropriate population for examining the development of writing attitudes, self-regulatory efficacy, and writing performance within the context of first-language (L1) writing education. Informed consent was obtained in accordance with ethical research principles (Cohen et al., 2018) on 14 May 2025 just before the commencement of the workshop. In total, 26 preservice Turkish language teachers participated in the quantitative strand of the study, comprising 10 male and 16 female students. Participants ranged in age from 18 to 22 years and were all enrolled in the third year of the Turkish Language Teacher Education program. The writing workshop was announced through the Faculty of Education, and participation was voluntary. Interested students were informed about the aims and procedures of the study before consenting to participate. These participants completed the pre-test and post-test measures of the Free Writing Attitude Scale and the Self-Regulatory Efficacy Scale for Writing. In addition, they produced final post-test writing tasks (informative and narrative texts), which were evaluated through rubric-based scoring.

For the qualitative strand, a subsample of 14 participants was identified using purposeful sampling strategies (Patton, 2015). Criteria for selection included gender balance, differences in writing performance as indicated by scale results, and varying levels of engagement and participation during the workshop activities. Such diversity enabled the exploration of both convergent and divergent perspectives, thereby enriching the qualitative data (Tisdell et al., 2025). The inclusion of both a broader quantitative sample and a smaller, strategically chosen qualitative subsample reflects the logic of mixed-methods research, in which general trends are complemented by in-depth contextualized insights (Creswell & Plano Clark, 2018).

The data collection process unfolded in three phases: pre-test administration, workshop implementation, and post-test administration with interviews. In the first phase, during the opening session of the workshop, all participants were briefed once again on the study’s aims and the voluntary nature of their involvement. They then completed the Free Writing Attitude Scale and the Self-Regulatory Efficacy Scale for Writing, providing baseline data on attitudes and self-regulation.

The second phase comprised the seven-week writing workshop, conducted by a team of university faculty members and professional writers. Each weekly session lasted three hours, with two hours devoted to theoretical content such as creative writing strategies, narrative construction, poetic forms, and editing practices and one hour to guided writing practice. Weekly assignments were given to reinforce learning, and students were encouraged to share and critique each other’s work. This intervention phase constituted the pedagogical treatment expected to influence participants’ writing-related competencies.

The final phase involved the post-test administration and qualitative interviews. At the conclusion of the workshop, participants once again completed the two scales and produced informative and narrative texts that were evaluated with analytic rubrics. To complement these quantitative measures, semi-structured interviews were conducted with the purposeful subsample of 14 participants. Interviews were carried out in a quiet setting, audio-recorded with consent, and transcribed verbatim for subsequent analysis. This qualitative strand provided deeper insight into participants’ experiences, challenges, and perceived benefits of the workshop.

Overall, the integration of multiple data sources (attitudinal scales, self-regulation measures, analytic assessments of final writing performance, and qualitative interviews) ensured methodological triangulation (Denzin, 2012). This comprehensive approach not only enhanced the reliability and validity of the findings but also aligned with best practices in mixed-methods research by capturing both measurable outcomes and lived experiences (Creswell & Plano Clark, 2018).

### 3.3. Data Collection Instruments

To investigate the effects of the writing workshop, the study employed multiple instruments that together captured the affective, cognitive, and performance dimensions of preservice teachers’ writing development. First, participants’ attitudes toward writing were measured using the Free Writing Attitude Scale developed by Ayrancı and Temizyürek (2017). This 19-item, 5-point Likert-type scale is organized into three dimensions: contribution to personal development, contribution to the development of writing, and contribution to social interaction. Representative items include “I believe that writing contributes to my personal development,” “I think that my writing ability can be improved,” and “I believe in sharing my writing with others.” Participants responded on a five-point scale ranging from 1 (strongly disagree) to 5 (strongly agree). The developers reported strong reliability indices, with Cronbach’s α values of 0.85, 0.83, and 0.73 for the respective sub-dimensions, and 0.88 for the overall scale. The scale thus provided a robust measure of the motivational and affective aspects of writing, which are known to be closely linked to writing success (Graham et al., 2007).

To complement the attitudinal data, participants’ self-regulatory capacities in writing were assessed using the Perceived Self-Regulatory Efficacy Scale for Writing. Originally developed by Zimmerman and Bandura (1994) within the framework of social cognitive theory and later adapted into Turkish by Çelikkaleli and Yıldırım (2015), the instrument consists of 25 items measuring learners’ efficacy beliefs in planning, monitoring, revising, and sustaining motivation during writing. Representative items include “I can begin writing without difficulty,” “I can remain focused on writing even when there are many distractions around me,” and “I can review the first draft of my writing and make necessary revisions.” Responses were recorded on a five-point scale ranging from 1 (strongly disagree) to 5 (strongly agree). Psychometric analyses of the Turkish adaptation demonstrated strong reliability (Cronbach’s α = 0.93) and a unidimensional structure explaining 38% of the total variance. Its theoretical grounding in self-regulated learning (Zimmerman, 2000) made it particularly suitable for capturing the metacognitive aspects of writing emphasized in the workshop.

To assess writing performance, participants completed two writing tasks at the conclusion of the workshop: one informative text and one narrative text. For the informative writing task, participants were asked to produce a structured expository text explaining and discussing a topic relevant to their experiences, knowledge, or interests. For the narrative writing task, participants were asked to compose an original story that included a coherent plot, characters, and sequence of events. Both tasks were completed under comparable assessment conditions, including identical time allocations, standardized instructions, and individual completion in the same instructional setting. The tasks were intended to assess participants’ ability to organize ideas, maintain coherence, use appropriate language, and communicate effectively across different genres. The resulting texts were evaluated using analytic rubrics adapted from Özdemir (2017). These rubrics assessed four key domains: page layout and title, spelling and punctuation, organization and coherence, and language and expression. Final post-test texts produced at the end of the workshop were evaluated independently by six raters, including three master’s graduates, two doctoral graduates, and one associate professor. The scores assigned by these raters were averaged to obtain the final performance scores, and inter-rater reliability was calculated to ensure objectivity, following established recommendations for rubric-based performance assessment (Stemler, 2004). The analytic scoring approach was deliberately chosen over holistic scoring, as it allows for more diagnostic feedback and a nuanced understanding of participants’ strengths and weaknesses in writing (Brookhart, 2013).

Finally, to gain deeper insight into participants’ experiences and perceptions, qualitative data were collected through semi-structured interviews conducted using a researcher-developed interview protocol. The interviews were carried out individually, audio-recorded with participants’ consent, and subsequently transcribed for analysis. The protocol contained six open-ended questions addressing perceived contributions of the workshop, challenges encountered, and changes in writing attitudes and self-regulation. Expert review was sought to establish content validity, and the interviews were conducted with a purposeful subsample of 14 students selected to ensure diversity in gender, baseline performance, and engagement (Patton, 2015). All interviews were audio-recorded with consent, transcribed verbatim, and later analyzed thematically in line with best practices in qualitative inquiry (Tisdell et al., 2025).

The integration of these instruments enabled the examination of writing development from multiple complementary perspectives. The Free Writing Attitude Scale captured participants’ affective orientations toward writing, the Self-Regulatory Efficacy Scale assessed their perceived capacity to regulate the writing process, the analytic rubrics provided objective evaluations of writing performance across informative and narrative genres, and the semi-structured interviews offered insight into participants’ experiences, perceptions, and self-reported writing practices. By combining quantitative and qualitative data sources, the study sought to provide a more comprehensive understanding of the cognitive, affective, and behavioral dimensions of writing development among preservice Turkish language teachers.

### 3.4. Data Analysis

The study employed a mixed-methods data analysis strategy to ensure that both the quantitative and qualitative dimensions of the research question were addressed comprehensively. Quantitative data were analyzed using the Statistical Package for the Social Sciences (SPSS) version 26.0. Descriptive statistics (means, standard deviations) were first calculated to provide an overview of participants’ performance on each measure. To examine changes between pre-test and post-test results, paired-sample *t*-tests were conducted. Because the same participants completed both measurement occasions, individual pre-test and post-test scores were matched using participant identifiers, enabling within-subject comparisons. Prior to analysis, normality was evaluated by examining skewness and kurtosis values. All values fell within acceptable ranges, indicating that the assumptions required for parametric testing were satisfied. Effect sizes were computed using Cohen’s *d* to determine the magnitude of observed changes (Cohen, 1988). In addition, inter-rater reliability for the rubric scoring of informative and narrative texts was assessed using Cohen’s kappa and intra-class correlation coefficients (ICCs), ensuring consistency and objectivity across raters (Stemler, 2004). Internal consistency reliability (Cronbach’s α) was recalculated for the scales in the current sample to confirm psychometric soundness in this specific context.

Qualitative data were drawn from the semi-structured interviews with 14 participants. All interview recordings were transcribed verbatim, and the transcripts were analyzed using inductive content analysis (Elo & Kyngäs, 2008). The process involved open coding, categorization, and abstraction to identify emergent themes reflecting participants’ experiences of the writing workshop. To enhance credibility and trustworthiness (Lincoln & Guba, 1985), multiple strategies were employed: (a) peer debriefing with two field experts to review coding decisions, (b) inclusion of direct quotations to illustrate themes, and (c) cross-checking of codes by a second researcher to establish inter-coder agreement.

Finally, integration of quantitative and qualitative findings was carried out during the interpretation stage, following the principles of triangulation (Denzin, 2012) and convergent parallel design (Creswell & Plano Clark, 2018). This allowed the study to corroborate statistically significant improvements in writing attitudes, self-regulation, and performance with participants’ personal accounts of growth, challenges, and perceived benefits, thus ensuring both breadth and depth in the research findings.

## 4. Findings

This section presents the findings of the study in relation to the research questions. Section 4.1 reports the quantitative findings addressing Research Questions 1–3, Section 4.2 presents the qualitative findings related to Research Question 4, and Section 4.3 integrates the quantitative and qualitative results to address Research Question 5.

### 4.1. Quantitative Findings (RQ1–RQ3)

#### 4.1.1. Free Writing Attitudes (RQ1)

The Free Writing Attitude Scale was administered to preservice teachers before and after the writing workshop to assess potential changes in their attitudes toward free writing. Descriptive statistics revealed an overall increase in mean scores from the pre-test (*M* = 62.13, SD = 8.74) to the post-test (M = 74.81, SD = 6.95). This represents a substantial improvement in participants’ attitudes following the intervention.

A paired-sample *t*-test was conducted to determine whether the observed difference was statistically significant. Results indicated a significant increase in free writing attitudes, *t*(25) = 5.42, *p* < .001, with a large effect size (*d* = 1.36), suggesting that the workshop had a strong positive impact on participants’ affective orientations toward writing. Table 1 presents the pre-test and post-test means, standard deviations, and test results.

Although the Free Writing Attitude Scale consists of three dimensions (personal development, development of writing, and social interaction), the present study employed the total scale score in the analyses. This decision was based on the study’s focus on overall attitudes toward free writing rather than dimension-specific differences and is consistent with previous applications of the scale. Accordingly, descriptive and inferential statistics are reported for the total scale score. Taken together, these findings demonstrate that participation in the seven-week writing workshop substantially improved preservice teachers’ attitudes toward free writing. This positive shift reflects findings from previous research indicating that sustained writing experiences and supportive writing environments can strengthen learners’ attitudes toward writing, increase confidence, and promote greater engagement in writing activities (Hall & Grisham-Brown, 2011; Bruning & Horn, 2000; Cremin & Oliver, 2017).

#### 4.1.2. Self-Regulatory Efficacy for Writing (RQ2)

The Self-Regulatory Efficacy Scale for Writing was administered before and after the workshop to assess participants’ perceived ability to plan, monitor, and regulate their writing process. Pre-test scores indicated a moderate level of self-regulatory efficacy (M = 71.44, SD = 7.12), while post-test scores reflected a marked improvement (M = 82.63, SD = 6.21).

A paired-sample *t*-test confirmed that this difference was statistically significant, *t*(25) = 6.03, *p* < .001, with a large effect size (*d* = 1.51). These results suggest that the workshop was effective in enhancing participants’ self-regulatory strategies for writing. Table 2 presents the comparison of pre-test and post-test scores on the self-regulatory efficacy scale:

#### 4.1.3. Writing Performance (RQ3)

Because informative and narrative writing samples were collected only at the conclusion of the workshop, the writing performance findings should be interpreted as descriptive indicators of participants’ writing performance at the end of the intervention rather than as direct evidence of improvement over time. Consequently, the results provide information about the quality and characteristics of participants’ final written products but do not permit pre–post comparisons of writing performance.

##### Informative Text Writing Performance (Rubric Scores)

Final post-test informative texts were evaluated using the analytic rubric, focusing on page layout and title, spelling and punctuation, organization and coherence, and language and expression. Table 3 presents descriptive statistics for participants’ overall informative writing performance scores as assessed using the analytic rubric. Because writing samples were collected only at the conclusion of the workshop, the writing performance assessment was intended to provide a summative evaluation of participants’ writing competence rather than a direct measure of growth over time. Consequently, unlike the attitude and self-regulation measures, the writing performance results should be interpreted as descriptive indicators of participants’ achievement at the end of the intervention rather than evidence of pre-post improvement:

##### Narrative Text Writing Performance (Rubric Scores)

Narrative texts were evaluated using the analytic rubric across the dimensions of page layout and title, spelling and punctuation, organization and coherence, and language and expression. Descriptive statistics for the overall narrative writing performance scores are presented in Table 4, providing a general indication of participants’ performance on the rubric-based assessment at the conclusion of the workshop. Overall narrative writing scores ranged from 56 to 74, with an average of M = 68.96 (SD = 4.28). The narrative writing scores exhibited some variation across participants, suggesting differences in their performance on the narrative writing task.

##### Inter-Rater Reliability of Writing Assessments

To ensure the objectivity of rubric-based text evaluations, inter-rater reliability was calculated for both informative and narrative texts. Six independent raters including three master’s graduates, two doctoral graduates, and one associate professor scored all texts. The scores were averaged to obtain final performance values, and consistency across raters was examined. For the informative texts, Cohen’s κ = 0.84 and the intra-class correlation coefficient (ICC) = 0.87, indicating high agreement. For the narrative texts, Cohen’s κ = 0.81 and ICC = 0.85, also reflecting strong reliability (Landis & Koch, 1977). These results demonstrate that the rubric-based scoring procedure was applied consistently across raters, thereby enhancing the reliability of the writing performance data. Table 5 presents the inter-rater reliability for writing performance assessments:

### 4.2. Qualitative Findings (RQ4)

The qualitative findings presented in this section address Research Question 4 by examining participants’ perceptions of the writing workshop and its influence on their writing-related experiences. To protect participants’ anonymity, pseudonymous codes were assigned to all qualitative responses. The letter “P” refers to “Participant,” and the accompanying number identifies the individual respondent (e.g., P4 = Participant 4). These codes are used throughout the presentation of the qualitative findings.

Thematic analysis of the semi-structured interviews revealed four major themes: perceived contributions of the writing workshop, challenges encountered, development of self-regulation, and attitudinal shifts toward writing. These themes provide nuanced insight into how participants experienced and interpreted the workshop, complementing the quantitative findings.

#### 4.2.1. Perceived Contributions of the Writing Workshop

The interview data revealed that preservice Turkish language teachers overwhelmingly perceived the writing workshop as a meaningful and transformative learning experience. Their reflections highlighted three main areas of contribution: development of writing competence, personal growth and confidence, and professional preparation as future teachers of the Turkish language.

Development of Writing Competence

Many participants emphasized that the workshop expanded their ability to generate ideas, structure texts, and refine stylistic elements such as vocabulary and sentence variety. They came to view writing as a skill that could be systematically developed rather than an innate gift. As one student reflected:

*“Now I think more comprehensively when I write; I look at events from a broader perspective. I diversified my reading, and I realized that writing is not simply a talent you are born with.”*

(P4)

Another participant expressed a similar realization:

*“At the beginning, I thought you had to be an expert to write well. This workshop showed me that we can make progress in writing by drawing on our everyday experiences.”*

(P1)

Personal Growth and Confidence

In addition to technical development, students consistently reported that the workshop enhanced their self-confidence and reduced their anxiety toward writing. Several noted that they overcame their prejudices against free writing and began to see it as a space for authentic self-expression. One participant explained:

*“I used to have negative feelings about free writing, but now it has become a form of freedom of expression. I can write comfortably, without hesitation, and in a more planned way.”*

(P12)

Another highlighted the impact on self-confidence:

*“I am more motivated now, and my confidence in myself has increased.”*

(P1)

Professional Preparation and Career Relevance

Several interviewees underlined that the workshop contributed directly to their future role as teachers. They emphasized that the strategies and techniques learned could be transferred to their own classroom practice. One participant stated:

*“I believe this will have a positive effect on my career. I learned how to organize the text I want to write and how to improve it further.”*

(P2)

Others valued the opportunity to interact with professional writers and academics, which they described as both inspiring and motivating for their own professional journeys.

Overall, the preservice teachers viewed the writing workshop as highly beneficial in shaping both their personal and professional identities as writers and future educators. They consistently reported that writing is a trainable and iterative process, not a fixed talent, and highlighted the workshop’s role in fostering competence, confidence, and readiness to model writing practices for their future students.

#### 4.2.2. Challenges Encountered During the Writing Process

Although the majority of participants highlighted the positive contributions of the workshop, they also identified several challenges they faced throughout the writing process. These challenges clustered around three main areas: time management and planning, linguistic and stylistic difficulties, and affective barriers such as self-confidence and anxiety.

Time Management and Planning

Several preservice teachers indicated that they struggled with effectively organizing their writing within the allocated time. While they acknowledged that planning strategies were introduced in the workshop, some found it difficult to balance idea generation, drafting, and revision. One participant noted:

*“I had many ideas in my mind, but I couldn’t manage my time well. Often, I had to finish my text quickly, and this made the ending weaker.”*

(P3)

Another echoed this concern, explaining:

*“I realized that if I do not plan carefully from the beginning, I lose too much time trying to decide what to write, and then I rush the rest.”*

(P8)

Linguistic and Stylistic Difficulties

Participants also reported challenges related to spelling, punctuation, and stylistic refinement. Some acknowledged gaps in their grammatical knowledge, while others struggled with maintaining coherence across longer texts. One student expressed:

*“Sometimes I couldn’t maintain consistency in my writing. The beginning was strong, but later on I lost control over the flow.”*

(P5)

Another pointed to stylistic concerns:

*“I had difficulty using diverse vocabulary and sentence structures. My writing felt repetitive.”*

(P11)

Affective Barriers: Self-Confidence and Anxiety

A smaller but notable group of students identified psychological barriers to writing. At the beginning of the workshop, some were hesitant to share their texts publicly or feared negative evaluation. This anxiety occasionally limited their engagement in group discussions. One preservice teacher explained:

*“At first, I was very nervous about reading my writing in front of others. I thought they would judge me or find my mistakes.”*

(P7)

Another described the emotional struggle as follows:

*“Sometimes I felt pressure to write perfectly. This made me anxious and blocked my creativity.”*

(P10)

Taken together, these findings show that preservice teachers not only gained skills and confidence but also encountered authentic difficulties typical of novice writers. Time management, language mechanics, and affective barriers emerged as significant obstacles. However, participants generally viewed these challenges as opportunities for growth, particularly as the workshop provided strategies to gradually overcome them.

#### 4.2.3. Development of Self-Regulation and Writing Habits

One of the most salient outcomes reported by participants was the development of self-regulation strategies and the gradual formation of more sustainable writing habits. Analysis of the interviews revealed three primary aspects of this development: planning and organization, monitoring and revising, and persistence in the writing process.

Planning and Organization

Students frequently noted that the workshop encouraged them to approach writing more systematically by engaging in pre-writing activities such as brainstorming, outlining, and setting goals. One participant described:

*“Before, I started writing without thinking much. Now, I first make a plan, decide what I want to say, and then follow that order while writing.”*

(P6)

Another emphasized the usefulness of planning for overcoming writer’s block:

*“When I get stuck, I go back to my outline. It helps me continue instead of stopping completely.”*

(P14)

Monitoring and Revising

Participants also reported that they became more attentive to monitoring the flow and coherence of their texts and revising drafts before finalizing them. Several indicated that revision, which they had previously neglected, became an integral part of their process. For example, one student explained:

*“I started to read my text again after finishing it. I check the meaning, the punctuation, and the connections between paragraphs. Revising used to feel like a burden, but now it feels natural.”*

(P15)

Another reflected on monitoring during the act of writing:

*“I try to pay attention to whether my sentences make sense as I write, not just after I finish. This prevents mistakes from accumulating.”*

(P9)

Persistence and Motivation

The workshop also helped participants sustain motivation and persist in writing despite challenges. Students described feeling more committed to completing tasks and more willing to write outside of class. One preservice teacher explained:

*“Even when I don’t feel like writing, I remind myself of the strategies we learned. It gives me discipline to continue.”*

(P2)

Another shared how self-regulation supported long-term habit formation:

*“I used to write only when it was necessary. Now I write more often, even for myself. It has become a habit.”*

(P13)

Overall, participants reported significant gains in their ability to plan, monitor, revise, and sustain effort in writing. These reflections align with the quantitative findings on self-regulatory efficacy, suggesting that the workshop not only improved perceptions of self-regulation but also translated into practical, observable strategies and writing habits.

#### 4.2.4. Attitudinal Shifts Toward Free Writing

The interviews demonstrated that the workshop had a profound impact on participants’ attitudes toward free writing. Many students reported that their initial hesitations, anxieties, or prejudices gave way to more positive, confident, and enthusiastic orientations. These shifts were evident across three dimensions: reduced anxiety and fear of mistakes, increased enjoyment and motivation, and recognition of free writing as a tool for self-expression.

Reduced Anxiety and Fear of Mistakes.

At the beginning of the workshop, some students described approaching free writing with hesitation, worrying about errors or lacking ideas. By the end, however, many explained that these concerns had diminished. One participant noted:

*“At first, I was afraid of making mistakes and felt pressured to write perfectly. Now, I don’t think about mistakes so much; I just focus on expressing myself.”*

(P10)

Another student explained:

*“Free writing used to feel stressful, but now I see it as a natural activity. I no longer think about what others will say about my text.”*

(P7)

Increased Enjoyment and Motivation

Participants emphasized that free writing had become more enjoyable and motivating. Instead of perceiving it as a forced task, they began to view it as a creative opportunity. One preservice teacher expressed:

*“I used to avoid writing because it felt like a duty. Now I enjoy it, and I even want to write outside of class.”*

(P12)

Another added:

*“The workshop changed my perspective. Writing is not boring anymore; it is something I look forward to.”*

(P3)

Recognition of Free Writing as Self-Expression

Students also highlighted that free writing became a means of personal expression and freedom, allowing them to connect their experiences, emotions, and perspectives with their writing. One participant commented:

*“Free writing has become a way to express myself. I write more honestly and without hesitation, and it reflects my personality.”*

(P1)

Another reinforced this view by stating:

*“I realized that free writing is not only about practicing skills; it is also about discovering yourself and your voice.”*

(P4)

In sum, participants described a clear attitudinal shift toward free writing, moving from anxiety and resistance to confidence, motivation, and self-expression. This transformation parallels the quantitative findings, which showed a significant increase in scores on the Free Writing Attitude Scale. Together, these results suggest that the workshop contributed to more positive attitudes toward writing and enhanced self-regulatory efficacy, as evidenced by the pre-test and post-test findings. In addition, participants reported perceived improvements in their writing practices and described a more positive affective relationship with writing. Because writing performance was assessed only at the end of the intervention, conclusions regarding changes in writing competence should be interpreted with appropriate caution.

### 4.3. Integration of Quantitative and Qualitative Findings (RQ5)

This section integrates the quantitative and qualitative findings to address Research Question 5 and provide a comprehensive understanding of participants’ experiences in the writing workshop. The integration of the quantitative and qualitative strands provides a comprehensive understanding of the experiences of preservice Turkish language teachers participating in the writing workshop. The quantitative findings demonstrate significant improvements in writing attitudes and self-regulatory efficacy, whereas the qualitative findings reveal participants’ perceptions of enhanced writing competence, confidence, and engagement. In addition, the rubric-based assessments provide complementary evidence regarding the quality and characteristics of participants’ final written products, thereby enriching the interpretation of the attitudinal and self-regulatory findings. Nevertheless, the convergence of quantitative and qualitative findings suggests that the workshop was associated with positive affective and self-regulatory outcomes and was perceived by participants as supportive of their writing development.

### 4.4. Convergence in Attitudes Toward Free Writing

Quantitative findings from the Free Writing Attitude Scale revealed a statistically significant increase in participants’ positive orientations toward writing (from *M* = 62.13 to *M* = 74.81). This numerical gain was directly supported by qualitative accounts, where students described moving from initial anxiety and reluctance to greater confidence, enjoyment, and motivation. For example, one participant emphasized that *“Free writing has become a way to express myself. I write more honestly and without hesitation”* (P1).

This convergence underscores two dimensions of change: not only did participants’ self-reported attitudes improve measurably, but their personal narratives illustrate how these changes were experienced affectively. In this way, the mixed-methods integration demonstrates that the workshop successfully addressed both the quantifiable outcomes of attitudinal growth and the lived transformation of writing as a more expressive, less stressful practice.

### 4.5. Convergence in Self-Regulation

Parallel findings were observed in the domain of self-regulation. The Self-Regulatory Efficacy Scale for Writing showed significant improvement (from *M* = 71.44 to *M* = 82.63), with a large effect size. Qualitative data substantiated these results by revealing the specific strategies participants adopted, including planning, monitoring, and systematic revision. As one student noted: *“Now, I first make a plan, decide what I want to say, and then follow that order while writing”* (P6).

The convergence of scale results with participant testimony suggests consistency between participants’ increased self-regulatory efficacy scores and their descriptions of writing practices adopted during the workshop. While the quantitative findings indicated greater confidence in self-regulation, the qualitative data provided insight into specific strategies participants reported using, including outlining, rereading, revising, and monitoring errors. Together, these findings suggest that participants perceived the workshop as supportive of the development of more deliberate and reflective writing practices during the intervention period.

### 4.6. Convergence and Divergence in Writing Performance

The rubric-based assessments of informative and narrative writing showed generally high levels of performance, with means of *M* = 69.69 (*SD* = 3.79) for informative texts and *M* = 68.96 (*SD* = 4.28) for narrative texts. While these scores indicate strong overall competence, qualitative accounts revealed a more nuanced picture. Students consistently identified informative writing as easier to structure and execute, whereas narrative writing presented greater challenges in creativity and coherence. One participant described: *“Sometimes I couldn’t maintain consistency in my stories. The beginning was strong, but later I lost control over the flow”* (P5).

This finding illustrates an important divergence between quantitative and qualitative strands. While the rubric scores alone suggest that narrative and informative writing were performed at similar levels, the qualitative reflections reveal that narrative writing was experienced as more demanding and less predictable. This divergence emphasizes the value of qualitative inquiry in uncovering dimensions of the writing process that may not be fully visible in numerical outcomes.

### 4.7. Triangulated Insights

When interpreted together, the quantitative and qualitative results present a cohesive picture of workshop effectiveness while also providing depth beyond statistical trends. Quantitative findings confirm statistically significant gains in writing attitudes and self-regulation, alongside strong performance outcomes. The qualitative narratives, however, reveal the emotional, cognitive, and social dynamics that shaped these gains, including initial anxieties, the adoption of new strategies, and the perceived empowerment that writing provided.

The mixed-methods triangulation highlights the complementary contributions of the quantitative and qualitative data sources. While the quantitative findings demonstrated significant gains in writing attitudes and self-regulatory efficacy, the qualitative findings provided insight into participants’ perceptions of their writing development and workshop experiences. The rubric-based assessments further offered descriptive information regarding participants’ writing performance at the end of the intervention. Nevertheless, the convergence of quantitative and qualitative findings suggests that participants experienced the workshop as supportive of both their writing development and their evolving identities as writers.

## 5. Discussion

The purpose of this study was to examine the effects of a writing workshop incorporating free writing activities on preservice teachers’ attitudes toward free writing, self-regulatory efficacy in writing, and writing performance across informative and narrative genres. Employing a convergent parallel mixed-methods design, the study sought to provide a comprehensive understanding of how structured yet flexible instructional environments influence both measurable outcomes and participants’ lived experiences of writing. The findings offer convergent evidence that writing workshops can function as powerful pedagogical spaces for fostering positive writing attitudes, strengthening self-regulation, and supporting writing development among preservice teachers.

### 5.1. Effects of the Writing Workshop on Free Writing Attitudes

The quantitative findings revealed a significant improvement in preservice teachers’ attitudes toward free writing following participation in the writing workshop. This result aligns with prior research suggesting that instructional practices emphasizing autonomy, expression, and reduced evaluative pressure can positively shape learners’ affective orientations toward writing (Elbow, 1973; Alharthi, 2021; Bruning et al., 2013; Anggraeni et al., 2025). By allowing participants to write without rigid constraints related to topic, genre, or correctness, free writing activities appear to have reframed writing as a generative and personally meaningful process rather than a performance-oriented task.

From a theoretical perspective, this shift can be interpreted as a movement away from product-oriented conceptions of writing toward process-oriented and writer-centered understandings, which have been shown to support sustained engagement and motivation (Graves, 1983; Graham & Perin, 2007). When learners perceive writing as an exploratory activity rather than a high-stakes evaluative task, they are more likely to approach writing with curiosity and willingness to take risks, both of which are central to positive writing attitudes (Petrić, 2002; Graham et al., 2007).

Qualitative findings further illuminate this shift, as participants frequently described free writing as liberating, motivating, and anxiety-reducing. These experiences are consistent with affective models of writing that emphasize the role of emotional engagement in sustaining writing behavior (Daly & Miller, 1975; Bruning & Horn, 2000). Reduced writing anxiety has been identified as a key factor in improving learners’ willingness to write and persist in writing tasks, particularly in contexts where previous writing experiences have been associated with fear of evaluation or error (Pollington et al., 2001; Aktaş & Akyol, 2020).

Importantly, the writing workshop context provided repeated opportunities for free writing, enabling participants to internalize positive writing experiences over time rather than encountering them as isolated activities. This sustained engagement may explain the robustness of the observed attitudinal changes. Research suggests that consistent exposure to supportive writing environments plays a crucial role in reshaping learners’ long-term beliefs and attitudes toward writing, as opposed to short-term interventions with limited transfer effects (Graves, 1983; Jasmine & Weiner, 2007). In this sense, the writing workshop functioned not only as an instructional setting but also as an affective space in which preservice teachers could reconstruct their relationship with writing.

### 5.2. Development of Self-Regulatory Efficacy in Writing

The study also demonstrated a significant increase in preservice teachers’ self-regulatory efficacy for writing. From a theoretical standpoint, this finding supports self-regulated learning models that conceptualize self-regulation as a skill that develops through iterative practice within supportive instructional environments (Zimmerman, 2000; Schunk & Zimmerman, 2007). The structure of the writing workshop—characterized by goal setting, drafting, feedback, revision, and reflection—naturally embedded opportunities for learners to engage in all three phases of self-regulation: forethought, performance, and self-reflection.

Within the context of writing instruction, such environments are particularly effective because they require learners to make continuous strategic decisions about content, organization, and language use, thereby fostering metacognitive awareness and control over the writing process (Hayes & Flower, 1980; Graham et al., 2012). As learners repeatedly engage in these cycles, self-regulatory behaviors become increasingly internalized, transforming writing from an externally guided task into a self-directed activity (Zimmerman & Bandura, 1994; Schunk & Zimmerman, 1998).

Participants’ qualitative accounts suggest that self-regulatory development was closely tied to increased awareness of their own writing processes. Many participants reported becoming more deliberate in planning their texts, monitoring their progress, and revising based on feedback. These findings resonate with empirical studies indicating that self-regulatory efficacy is strengthened when learners perceive writing as manageable and controllable rather than overwhelming (Graham et al., 2012; Festas et al., 2015). Similar patterns have been observed in studies emphasizing formative feedback and reflective practice, where learners’ sense of agency in writing tasks contributes directly to improved self-regulatory beliefs (Pollington et al., 2001; M. F. Teng et al., 2022).

Notably, the integration of free writing within the workshop may have played a facilitative role in this process. By reducing affective barriers and encouraging risk-taking, free writing likely created conditions under which participants felt more confident experimenting with strategies and reflecting on their writing behaviors. This finding extends previous research by suggesting that affective engagement may serve as a precursor to effective self-regulation in writing. When learners experience writing as emotionally safe and personally meaningful, they are more willing to engage in self-monitoring and revision, which are central mechanisms of self-regulated learning (Daly & Miller, 1975; Bruning & Horn, 2000; Petrić, 2002).

### 5.3. Effects on Writing Performance Across Genres

In addition to the attitudinal and self-regulatory findings, the study provided descriptive evidence regarding participants’ writing performance in informative and narrative genres at the conclusion of the workshop. The analytic rubric assessments indicated that participants were generally able to produce organized, coherent, and technically appropriate texts across both genres. Although these findings offer insight into participants’ writing performance following the intervention, writing samples were not collected prior to the workshop; therefore, the results should not be interpreted as direct evidence of improvement in writing competence. Instead, the writing assessments complement the quantitative and qualitative findings by providing an additional perspective on participants’ writing outcomes at the end of the seven-week workshop. While direct conclusions regarding improvement cannot be drawn from the present design, the overall pattern of findings is broadly consistent with research suggesting that process-oriented and workshop-based writing instruction can support writing development and engagement (Graham & Perin, 2007; Berne, 2009).

From a cognitive-process perspective, such improvements suggest that participants developed greater control over higher-order composing skills, including planning, translating ideas into text, and revising for clarity and coherence (Hayes & Flower, 1980; Hayes, 2012). Research indicates that instructional approaches which foreground recursive writing processes and formative feedback are particularly effective in promoting these higher-level skills across different writing contexts (Graham et al., 2012; De Smedt & Van Keer, 2018).

The genre-based findings suggest that participants were able to engage with both informative and narrative writing tasks within the workshop context. Although the present study does not provide direct evidence regarding the transfer of writing skills across contexts or genres, the qualitative findings indicate that participants perceived process-oriented strategies such as planning, monitoring, revising, and reflection as useful across different writing tasks. These perceptions are particularly relevant within the context of preservice Turkish language teacher education, where future language teachers are expected to develop writing competence and to support writing development in their future classrooms. The findings therefore suggest that writing workshops may provide opportunities for preservice language teachers to engage with writing as a process rather than as a set of genre-specific procedures. This interpretation is consistent with scholarship emphasizing the value of procedural knowledge and strategic writing processes in supporting writers’ adaptability across writing situations (Hyland, 2004; Beaufort, 2007).

The finding that participants reported using planning, monitoring, revising, and reflective strategies across both informative and narrative writing tasks aligns with research emphasizing the central role of self-regulation in writing development (L. S. Teng, 2022; Hwang, 2025). Rather than viewing writing as a product-oriented activity, participants appeared to engage with writing as a recursive process involving goal setting, self-monitoring, and revision, which are considered key characteristics of effective writers (Zimmerman & Bandura, 1994; Hayes, 2012). The qualitative differences reported between informative and narrative writing are also consistent with genre-based research suggesting that narrative writing places greater demands on creativity, audience engagement, and coherence management, whereas informative writing tends to benefit from more explicit organizational structures (Hyland, 2004; Beaufort, 2007). Taken together, these findings suggest that writing workshops may provide a supportive context for fostering process-oriented writing practices that can be applied across different writing genres, although future studies employing pre- and post-intervention writing assessments are needed to examine changes in writing performance more directly.

### 5.4. Implications for Practice and Policy

The findings of this study offer several important implications for educational practice and policy, particularly within the context of preservice Turkish language teacher education and writing instruction. The quantitative findings demonstrated significant positive changes in participants’ writing attitudes and self-regulatory efficacy, while the qualitative findings suggested that participants perceived the writing workshop as supportive of their writing development, confidence, and engagement. In addition, the rubric-based assessments provided descriptive information regarding participants’ writing performance at the conclusion of the intervention. Collectively, these findings highlight the potential value of instructional environments that integrate cognitive, affective, and self-regulatory dimensions of writing development.

### 5.5. Implications for Preservice Language Teacher Education

The findings of this study have several implications for the preparation of preservice Turkish language teachers within first-language (L1) writing education contexts. The significant improvements observed in writing attitudes and self-regulatory efficacy suggest that writing workshops may provide a valuable complement to traditional writing instruction by creating opportunities for sustained writing practice, reflection, and strategic engagement with the writing process. Consistent with research on self-regulated learning and writing development (Bruning & Horn, 2000; Zimmerman, 2000; L. S. Teng, 2022), instructional environments that encourage planning, monitoring, revision, and reflection may help future language teachers develop greater confidence in their own writing practices.

The qualitative findings further suggest that participants perceived free writing activities as supportive of engagement, expression, and reduced apprehension toward writing. Incorporating regular low-stakes writing opportunities within language teacher education programs may therefore encourage greater participation in writing activities and provide opportunities for preservice teachers to experience process-oriented writing pedagogy firsthand. Such experiences are particularly relevant for future language teachers, whose own writing experiences may influence their subsequent approaches to writing instruction (Hall & Grisham-Brown, 2011; Morgan, 2010; Cremin & Oliver, 2017).

More broadly, the findings support the inclusion of structured opportunities for writing, reflection, and feedback within language teacher education curricula. Although the present study does not provide direct evidence regarding long-term effects or improvements in writing competence over time, the positive attitudinal, self-regulatory, and qualitative outcomes suggest that writing workshops represent a promising pedagogical approach worthy of further investigation within L1 language teacher education contexts.

### 5.6. Limitations and Directions for Future Research

Despite its contributions, the study has several limitations that should be acknowledged. First, the sample was limited to preservice teachers from a single institutional context, which may constrain the generalizability of the findings. Future research could replicate the study across diverse educational settings and cultural contexts. Second, the duration of the intervention was relatively short, and no follow-up data were collected after the completion of the workshop. Consequently, although participants reported positive changes in their attitudes toward writing and self-regulatory practices during the intervention period, the study cannot determine whether these effects were sustained over time. Longitudinal research is therefore needed to examine the persistence and long-term development of attitudinal and self-regulatory outcomes following participation in writing workshops.

An additional limitation concerns the assessment of writing performance. Although informative and narrative texts were evaluated using analytic rubrics, writing samples were collected only at the end of the intervention. Consequently, the study was unable to examine changes in writing performance over time or directly attribute observed levels of writing competence to the workshop. Future research should incorporate pre-test and post-test writing assessments to provide stronger evidence regarding the effects of writing workshops on writing performance development.

Future studies might also explore the differential impact of specific workshop components, such as peer feedback or reflective writing, on self-regulation and performance. Additionally, examining how preservice teachers transfer their workshop experiences into classroom practice would provide valuable insight into the long-term pedagogical impact of writing workshops.

## 6. Conclusions

In conclusion, the present study provides robust evidence that writing workshops incorporating free writing activities can positively influence preservice teachers’ writing attitudes, self-regulatory efficacy, and writing performance. By integrating affective, cognitive, and behavioral dimensions of writing development, the study contributes to the growing body of international research advocating for process-oriented, learner-centered writing pedagogy. The findings position writing workshops not only as instructional tools for skill development but also as transformative spaces for cultivating reflective, self-regulated, and confident writers in teacher education.

## Figures and Tables

**Table 1:** Comparison of Pre-test and Post-test Scores on the Free Writing Attitude Scale.

Measure	Pre-Test M (SD)	Post-Test M (SD)	*t*	*p*	Cohen’s *d*
Free Writing Attitude Scale	62.13 (8.74)	74.81 (6.95)	5.42	<.001	1.36

**Table 2:** Comparison of Pre-test and Post-test Scores on the Self-Regulatory Efficacy Scale.

Variable	Pre-Test Mean (SD)	Post-Test Mean (SD)	t	*p*	Effect Size (*d)*
Self-Regulatory Efficacy for Writing	71.44 (7.12)	82.63 (6.21)	6.03	<.001	1.51

**Table 3:** Descriptive Statistics for Informative Text Scores.

Measure	M	SD	Min	Max
Informative	69.69	3.79	57	74

**Table 4:** Descriptive Statistics for Narrative Text Scores.

Measure	M	SD	Min	Max
Narrative	68.96	4.28	56	74

**Table 5:** Inter-rater Reliability for Writing Performance Assessments.

Text Type	Cohen’s κ	ICC	Interpretation
Informative	0.84	0.87	High agreement
Narrative	0.81	0.85	High agreement

## Data Availability

The data presented in this study are available on reasonable request from the corresponding author. The qualitative data are not publicly available due to the risk of decontextualization.
